# *In vivo* selection of tumor-specific antibodies

**DOI:** 10.18632/oncotarget.1407

**Published:** 2013-09-20

**Authors:** David Sánchez-Martín, Laura Sanz, Erkki Ruoslahti, Luis Alvarez-Vallina

**Affiliations:** Molecular Immunology Unit, Hospital Universitario Puerta de Hierro Majadahonda, 28222 Majadahonda, Madrid, Spain

One of the most promising strategies for the development of effective cancer therapies relies on the targeted delivery of antibody-based therapeutics. Effective tumor targeting with antibodies depends on the identification of new targets, and the optimization of antibody structure [1, 2]; however, the discovery and validation of novel tumor-associated antigens remains challenging [1]. Indeed, the FDA-approved antibodies for cancer indications are directed against a limited number of targets. Often, novel antibodies are raised against well-known antigens, trying to surpass pre-existing ones in favorable properties. This approach can be helpful; for example, the upcoming obinutuzumab seems to outperform the blockbuster rituximab [3], but it doesn't necessarily imply an advance in our understanding of the disease. In contrast, using a functional approach, it is possible to select monoclonal antibodies with a defined biological effect that can lead to the identification of new or less well studied proteins potentially involved in the pathogeny of diseases [4]. This strategy has greater potential for innovation and can be used to address the unmet need in oncology of discovering systemically accessible antigens preferentially overexpressed in the tumor microenvironment.

We have recently used a functional approach to identify antibodies able to deliver a payload to the primary tumor in a xenograft mouse model [5]. Using a highly diverse (~3 × 10^9^) combinatorial library of single domain antibodies ensures the presence of antibodies against any potentially relevant target. We performed the selection *in vivo*—letting the antibody library circulate in the mouse—and selected only those antibodies that met the defined biological effect, without previous knowledge of the target. This unbiased approach can yield new antigens, discriminates against antibodies with unexpected off-target effects; and emphasizes the availability of the epitope *in vivo*. This latter aspect is sometimes overlooked when selecting antibodies for therapy, as antibodies selected using functional screens *in vitro* can fail in the clinic because of the *in vivo* environment is different. One of the antibodies selected with our new strategy recognizes the proteasome activator complex PA28. We found that the expression of the α subunit of PA28 is elevated in primary and metastatic human prostate cancer and used anti-PA28α antibodies to show that PA28 is accessible in mouse xenograft tumors. These results support the use of PA28 as a tumor marker, and potentially, as a target for therapeutic intervention in prostate cancer.

We have focused on antibody discovery in tumor-bearing mice; however, this procedure is applicable to any disease for which an animal model exists (be it cancer, neurodegenerative diseases, etc.). Furthermore, the phage display technology can be used to study not only antibodies, but also to investigate any other extracellular protein-protein interactions [6, 7] *in vivo*, and to map such interaction in the organism where they occur.
